# Virtual screening of a random tripeptide library for easily prepared inhibitors of insect chitinolytic enzymes

**DOI:** 10.1016/j.tcsw.2025.100143

**Published:** 2025-05-13

**Authors:** Zihan Pang, Yi Ding, Huijie Xie, Xi Jiang, Tian Liu

**Affiliations:** a MOE Key Laboratory of Bio-intelligent Manufacturing, School of Bioengineering, Dalian University of Technology, Dalian 116024, China; b College of Life and Health, Dalian University, Dalian, Liaoning 116622, China

**Keywords:** Insect chitinolytic enzymes, Tripeptide inhibitors, Virtual screening, Molecular dynamics simulations

## Abstract

Insect molting requires chitinolytic enzymes to hydrolyze the chitin in the old cuticle, making chitinolytic enzyme inhibitors potential insecticides. Naturally occurring peptide chitinase inhibitors, such as argadin and argifin, possess complex non-natural structures, making large-scale production and application difficult. Given that chitinolytic enzymes recognize a core trisaccharide unit, this study integrates both in silico and in vitro approaches to explore the feasibility of using tripeptides as insect chitinolytic enzyme inhibitors. Virtual screening of 8000 random tripeptides identified 202 candidate peptides, which were found to be more hydrophobic and enriched in aromatic amino acids, complementing the properties of the chitinase substrate-binding pocket residues. Four selected peptides were synthesized and their inhibitory activities against chitinolytic enzymes from *Ostrinia furnacalis* were quantitatively assessed, with QWW exhibiting an IC_50_ value of 0.2 mM against *Of*Chi-h. Molecular dynamics simulations indicated that strong hydrogen bonds and π-π interactions were key factors in the high activity of QWW. This study not only expands the chemical space for agricultural chemicals targeting chitinase but also establishes an extendable, peptide-based inhibitor discovery process, which may apply to drug development for other targets.

## Highlights

• Insect chitinolytic enzymes are potential multitargets for designing pesticides.
• Tripeptides' modular diversity enables novel inhibitor discovery via in silico screening.
• QWW tripeptide showed potent inhibitory activity via H-bonds and π-π interactions.

## 1 Introduction

Chitin, an essential structural polysaccharide composed of *N*-acetyl-β-d-glucosamine (GlcNAc) units linked by *β*-1,4-glycosidic bonds, is a fundamental component of the insect cuticle. In association with cuticular proteins, it forms a robust and flexible composite that provides mechanical support and serves as a protective barrier against chemical agents, physical abrasion, and microbial invasion (Qu et al., 2021). However, the rigidity of the chitin-based exoskeleton imposes considerable constraints on body expansion. To accommodate growth and development, insects undergo periodic molting, during which the old cuticle is enzymatically degraded and subsequently rebuilt. This process is orchestrated by a chitinolytic enzyme system secreted by epidermal cells, allowing for exoskeletal remodeling and morphological renewal (Chen et al., 2018).

Chitinases (EC 3.2.1.14) are classified within glycoside hydrolase family 18 (GH18) and are primarily responsible for the enzymatic degradation of chitin, a key structural polysaccharide in insect exoskeletons. GH18 chitinases can be further subdivided into several subgroups, including ChtI, ChtII, and Chi-h, each of which fulfills a distinct and indispensable physiological function during insect metamorphosis and development. In addition to GH18 chitinases, insects also express *β*-*N*-acetyl-d-hexosaminidase (Hex1, EC 3.2.1.52) belonging to glycoside hydrolase family 20 (GH20), which acts synergistically with chitinases to degrade chitin in the old cuticle (Qu et al., 2014). Notably, both GH18 chitinases and GH20 Hex enzymes employ a substrate-assisted catalytic mechanism and exhibit comparable substrate-binding modes, rendering them promising targets for the development of multi-target insecticidal inhibitors (Ding et al., 2023b). Accumulating evidence has underscored the pivotal role of GH18 chitinases in insect growth and development, particularly in mediating chitin degradation during molting. Among these enzymes, ChtII demonstrates high catalytic efficiency toward crystalline chitin and facilitates the subsequent activity of auxiliary enzymes (Chen et al., 2018), whereas ChtI preferentially targets amorphous or intermediate forms of chitin (Dong et al., 2019). Accordingly, inhibition of these enzymes disrupts the molting process and frequently results in developmental arrest or insect mortality. Due to their essential roles in insect physiology and molting, chitinolytic enzymes have been recognized as promising molecular targets for the development of environmentally sustainable pest control agents (Ding et al., 2023b; Ding et al., 2023a; Lu et al., 2023).

Peptides, composed of short chains of amino acids linked by peptide bonds, have attracted increasing interest due to their wide applications in biomedicine (Liu et al., 2025; Mu et al., 2024), materials science (Kaygisiz et al., 2025; Li et al., 2024), and the development of enzyme inhibitors (Kiefer et al., 2025; Van Wier and Beekman, 2025). Their modular structure and rich chemical diversity provide advantages for discovering novel molecular recognition mechanisms. Several studies have identified peptides as potent chitinase inhibitors, showing great potential in this field. For instance, natural cyclic peptides such as argifin and argadin mimic the binding mode of chitin oligosaccharides and inhibit chitinase activity at micromolar to nanomolar concentrations (Dixon et al., 2006). However, their structural complexity, synthesis difficulty, and high production costs, along with the limited amino acid diversity in their native scaffolds, hinder further optimization and drug development, limiting broader use in insect control.

To overcome the limitations of existing chitinase inhibitors in terms of structural complexity and synthetic difficulty, this study presents a novel approach: a combinatorial library of 8000 tripeptides was constructed by random assembly of natural amino acids to identify tripeptides with inhibitory activity against *O. furnacalis* chitinolytic enzymes. Based on the affinity calculated by molecular docking, 202 candidate peptides were identified. Sequence analysis of candidate peptides and non-candidate peptides revealed that peptides with higher hydrophobicity and rich aromatic amino acids are better suited to the chemical properties of the substrate-binding pocket, suggesting a potential common feature of chitinase inhibitors. Among the screened candidates, the QWW tripeptide (Gln-Trp-Trp) showed potent inhibitory activity against insect chitinase with an IC₅₀ value in the micromolar range. Unlike argifin and argadin derivatives, the QWW tripeptide scaffold simplifies the synthesis process by avoiding the complexity of cyclic structures. Molecular dynamics simulations revealed that strong hydrogen bonds and π-π interactions are key factors contributing to the high activity of these peptides. In addition, the tripeptide scaffold provides a versatile platform for further optimization, such as the incorporation of non-canonical amino acids or functionalized side chains. This study not only expands the chemical space for agrochemical development targeting chitinolytic enzymes but also establishes a scalable, peptide-based inhibitor discovery pipeline that can be applied to other enzyme targets. Importantly, this workflow enables even non-specialist chemists to readily access easily-prepared peptide inhibitors for their target enzymes.

## 2 Materials and methods

### 2.1 Materials

The 4-Methylumbelliferyl-*β*-D-*N*,*N*′-diacetylchitobiose [MU-(GlcNAc)_2_] and 4-methylumbelliferyl-*β*-D-*N*-acetylglucosaminide (MU-GlcNAc) were purchased from Sigma-Aldrich (Shanghai, China). All tripeptides were synthesized by Bio-Technology (Shanghai, China).

### 2.2 Construction of the tripeptide library

To construct the tripeptide library, we first utilized a Python script to generate 8000 random linear tripeptide sequences in FASTA format. These sequences were then converted to SDF files using the RDKit toolkit (https://www.rdkit.org) for further structural processing. The MMFF94 force field (Halgren and Nachbar, 1996) was applied to minimize the energy of each tripeptide molecule, ensuring the generation of reasonable three-dimensional conformations. This diverse set of tripeptide structures will be used for subsequent molecular docking and screening analyses.

### 2.3 Molecular docking

All tripeptide ligands were preprocessed using MGLTools, including water removal, hydrogen addition, and charge assignment. Structural data for four enzymes were downloaded from the RCSB website: *Of*Chi-h (PDB ID: 5GQB) (Chen et al., 2018), *Of*ChtI (PDB ID: 3WQW) (Liu et al., 2017), *Of*ChtII (PDB ID: 5Y29) (Chen et al., 2014), and *Of*Hex1 (PDB ID: 3NSN) (Liu et al., 2011), and preprocessed using MGLTools. The docking box dimensions were set as follows: *Of*Chi-h (50 × 50 × 50 Å), *Of*ChtI (126 × 70 × 82 Å), *Of*ChtII (50 × 60 × 66 Å), and *Of*Hex1 (50 × 50 × 50 Å). Molecular docking was carried out using two software tools: AutoDockVina (Eberhardt et al., 2021; Trott and Olson, 2010) and LeDock (Wang et al., 2016). For AutoDockVina, the Lamarckian genetic algorithm was applied, conducting 25 million energy evaluations over 27,000 generations. In parallel, LeDock was used with default parameters, where conformation sampling was performed using a combination of simulated annealing and evolutionary optimization. Docking scores from both methods were calculated using the respective default scoring functions. From the combined docking scores, the top 500 scoring tripeptides for each enzyme were selected. After taking the intersection of these sets, a total of 202 tripeptides were identified as candidate molecules.

### 2.4 Amino acid sequence analysis

The sequence characteristics of candidate molecules were analyzed using a Python script, and the following parameters were calculated: proportion of aromatic amino acids, proportion of hydrophobic amino acids, grand average of hydropathicity (GRAVY), proportion of cationic amino acids, and proportion of anionic amino acids. GRAVY is defined as the sum of the hydrophobicity values of all amino acids in the sequence divided by the total number of amino acids. Negative GRAVY values indicate stronger hydrophilicity, while positive values indicate higher hydrophobicity. The mean and standard deviation of the proportions of aromatic amino acid composition, the proportion of hydrophobic residues, the GRAVY values, and the proportions of positively and negatively charged amino acids were obtained by calculating the parameters for each molecule of the 202 candidate tripeptides and the remaining tripeptides.

### 2.5 Enzyme preparation

Insect chitinases, including *Of*ChtI, *Of*ChtII, *Of*Chi-h, and *Of*Hex1 from *O. furnacalis*, were expressed in *Pichia pastoris* GS115 and purified as described previously (Chen et al., 2014, Chen et al., 2018; Liu et al., 2015; Liu et al., 2013). The purities of the target proteins were analyzed by sodium dodecyl sulfate-polyacrylamide gel electrophoresis.

### 2.6 Inhibitory activity assay

The final volume of the reaction mixture used for the inhibitor screening was 100 μL, consisting of the enzyme, 10 μL of substrate (the substrate for *Of*ChtI, *Of*ChtII, and *Of*Chi-h was MU-(GlcNAc)_2_, and the substrate for *Of*Hex1 was MU-GlcNAc) in 20 mM sodium phosphate buffer (pH 6.0) containing 2 μL of inhibitor. The final concentration of substrate was 10 μM. A reaction without inhibitor served as the control. After incubating at 30 °C for 30 min, 100 μL of 0.5 M Na_2_CO_3_ was added to terminate the reaction. The fluorescence of the released 4-methylumbelliferone was quantified using an Infinite200 PRO microplate reader (Tecan, Switzerland), with excitation and emission at 360 and 450 nm, respectively.

### 2.7 Molecular dynamics simulations

Atomistic molecular dynamics (MD) simulations were conducted using GROMACS 2022.4 (Abraham et al., 2015), applying the amber99sb-ildn force field (Lindorff-Larsen et al., 2010). The initial conformations for the MD simulations were generated with LeDock, a docking software tool specifically designed for predicting ligand binding poses to receptors. To ensure accurate electrostatic calculations for the ligands, RESP2 charges were calculated (Helmich-Paris et al., 2021). These partial charges were derived using the ORCA (Helmich-Paris et al., 2021; Izsák et al., 2013; Izsák and Neese, 2011; Neese, 2022; Neese et al., 2009) program, a quantum chemistry software package, in conjunction with Multiwfn (Lu, 2024; Lu and Chen, 2012), a tool used for wavefunction data analysis and visualization. The RESP2 method, a variant of the Restrained Electrostatic Potential approach, is widely employed to derive atomic charges consistent with quantum mechanical calculations, thus providing a more accurate representation of ligand-receptor interactions during the MD simulations. The selected docking complexes were solvated using the TIP3P water model. A cubic simulation box was created to calculate the protein-ligand interactions, with the box size chosen to minimize interactions through periodic boundaries. Nonbonded interactions were truncated at a cutoff distance of 8 Å. The system was initially equilibrated using the steepest descent method for 5000 steps, with the protein and ligand atoms restrained at 10 kcal/mol and 0 kcal/mol, respectively. Subsequently, the system was gradually heated to 298 K over 50 ps, maintaining a 20 kcal/mol restraint on the protein-ligand complex. Following this, a 1 ns isothermal-isobaric (NPT) ensemble simulation and a 1 ns canonical (NVT) ensemble simulation were performed under a 5 kcal/mol restraint. Finally, a 100 ns MD run was performed for further equilibration and sampling. All MD simulations were conducted with 2 fs time steps, with temperature control achieved using the Berendsen thermostat. The protein-ligand complexes were deemed well-equilibrated based on the monitoring of the root-mean-square deviation (RMSD) of the ligand and protein backbones. Stable and equilibrated ligand conformations were extracted from the final nanoseconds of the MD simulation.

### 2.8 Decomposition of binding free energy

To comprehensively elucidate the mechanisms underlying protein-ligand interactions, the molecular mechanics/Poisson–Boltzmann surface area (MM/PBSA) method (Valdés-Tresanco et al., 2021) was employed to decompose the binding free energy. This approach evaluates the contributions of electrostatic interactions, van der Waals forces, polar solvation energy, and nonpolar solvation energy to the overall binding free energy. Specifically, a set of 2000 representative snapshots was generated by extracting frames at 1 ps intervals from the last 50 ns of the 100 ns molecular dynamics simulation trajectory, ensuring comprehensive sampling and accurate characterization of the system.

## 3 Results

### 3.1 Tripeptide library construction and screening for chitinase inhibitors

**Fig. 1:**
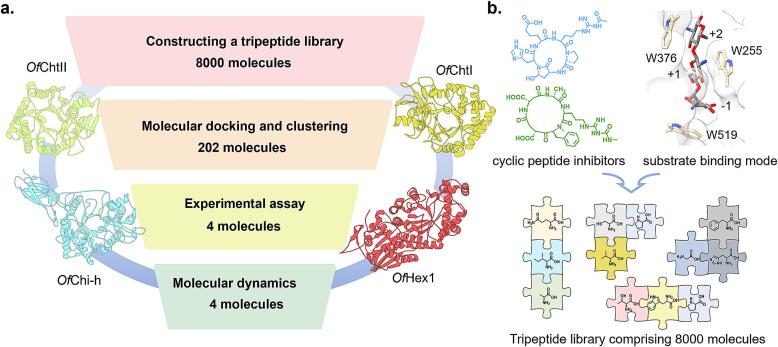
(a) Virtual screening workflow for tripeptide inhibitors targeting insect chitinolytic enzymes. (b) Construction strategy for an easily synthesizable tripeptide library.

Given that the minimal catalytic unit of chitinases is a trisaccharide, this study focused on tripeptides to simplify the design of natural cyclic peptide inhibitors of chitinases (Fig. 1). Compared to natural cyclic peptides, the tripeptide library not only simplifies the structure but also provides superior synthetic feasibility. By diversifying the amino acid composition, tripeptides further enhance both the structural and functional diversity of chitinase inhibitors.

To construct the tripeptide library, we first randomly arranged and combined 20 natural amino acids, resulting in a total of 8000 possible tripeptides (Fig. 1b). Subsequently, we retrieved the FASTA sequences of all tripeptides and used the RDKit toolkit to convert them into SDF files containing three-dimensional coordinates. These structures were subjected to energy minimization to ensure that each tripeptide adopted a reasonable three-dimensional conformation.

To evaluate the binding affinity between peptides and four chitinolytic enzymes (*Of*ChtI, *Of*ChtII, *Of*Chi-h, and *Of*Hex1), molecular docking was conducted. Based on the docking scores, the top 500 scoring tripeptides for each enzyme were selected. The intersection of these sets resulted in a total of 202 candidate molecules, which provided a potential molecular basis for subsequent enzyme inhibition research.

### 3.2 Identification of potent tripeptide inhibitors based on sequence analysis of candidate molecules

**Fig. 2:**
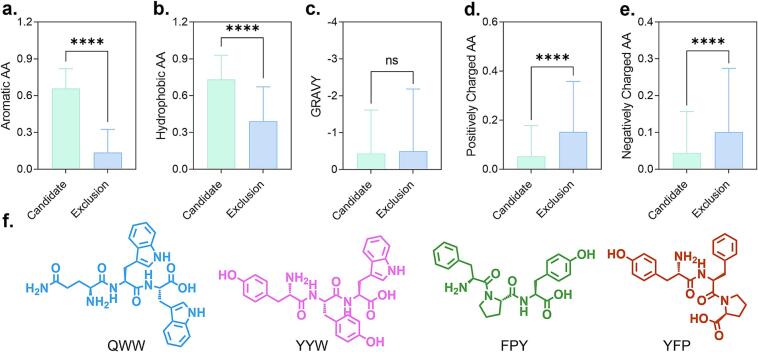
Properties of the 202 candidate tripeptides and the remaining sequences are shown, including: (a) proportion of aromatic amino acids, (b) proportion of hydrophobic amino acids, (c) grand average of hydropathicity (GRAVY), (d) proportion of positively charged amino acids, and (e) proportion of negatively charged amino acids. (f) Chemical structures of the tripeptides QWW, YYW, FPY, and YFP.

Statistical analysis of the 202 candidate tripeptides revealed a significant enrichment of aromatic amino acids, particularly tryptophan, phenylalanine, and tyrosine. These residues were frequently located at all three positions of the tripeptides, with tryptophan most commonly appearing at the C-terminal position (Fig. 2a). This pattern was not observed among the non-candidate sequences, suggesting a potential structural preference for aromatic residues during the selection process. Moreover, the candidate tripeptides exhibited a significantly higher proportion of hydrophobic amino acids (Fig. 2b), which is consistent with the hydrophobic character of the substrate-binding pockets of chitinases. Although no statistically significant difference in GRAVY (grand average of hydropathicity) values was observed between the candidate and non-candidate tripeptides (Fig. 2c), the candidate set generally exhibited lower hydrophilicity. This observation may be attributed to the counteractive effects of nonpolar aromatic side chains of tryptophan, phenylalanine, and tyrosine on hydrophilicity metrics in GRAVY calculations. Therefore, lower GRAVY values may serve as an indirect indicator of aromatic residue enrichment and support the structural compatibility of these peptides with the hydrophobic microenvironment of chitinase binding pockets. Further compositional analysis (Fig. 2d and e) revealed that candidate tripeptides contained significantly lower proportions of both positively and negatively charged residues. This pattern may reflect adaptation to the electrostatic properties of the chitinase active sites. All three chitinases from *O. furnacalis*, namely *Of*ChtI, *Of*ChtII, and *Of*Chi-h, share the conserved catalytic sequence DxDxE, which is essential for proton donation and nucleophilic activation in glycosidic bond cleavage. In addition, arginine residues are frequently distributed around the substrate-binding region and are likely involved in substrate recognition and stabilization through hydrogen bonding and electrostatic interactions. Tripeptides with excessive charged residues may introduce redundant hydrogen bond donors or acceptors, disturb local electrostatic complementarity, and reduce their adaptability to different targets. In contrast, tripeptides with a limited number of spatially compatible polar residues may retain favorable binding affinity and exhibit broader compatibility across multiple chitinases.

During the selection of representative tripeptides, the high frequency of tryptophan at the third position was particularly noteworthy. While its aromatic and hydrophobic properties facilitate π–π interactions and hydrophobic interactions within the enzyme pocket, the presence of multiple bulky aromatic side chains may lead to steric hindrance, hindering optimal accommodation. To investigate the binding capacity of aromatic-rich sequences, YYW was selected as a representative tripeptide due to its potential for extensive π–π interactions and hydrogen bonding. QWW was additionally selected as a comparative sequence, incorporating glutamine at the N-terminal position. As a polar but uncharged residue, glutamine was expected to enhance interactions with polar side chains within the binding environment. Structural analysis of multiple chitinases revealed that the substrate-binding subsites from position −1 to +2 are frequently flanked by three tryptophan residues, forming a spatially favorable aromatic cavity that could support the binding of aromatic-enriched tripeptides. Further statistical analysis identified proline as one of the most frequently occurring non-aromatic residues among the candidate sequences. Due to its rigid cyclic structure, proline imposes conformational constraints on the peptide backbone, thereby stabilizing local geometry. Based on this observation, FPY and YFP were selected as representative structures. In FPY, proline is located in the central position, contributing to a compact, sandwich-like conformation. In YFP, proline occupies the C-terminal position, allowing increased flexibility at the C-terminus, particularly for the terminal tyrosine. These structural arrangements provide a rational framework for further investigating the relationship between backbone rigidity and chitinase inhibitory activity.

### 3.3 Inhibitory activity of selected tripeptides against chitinolytic enzymes

**Fig. 3:**
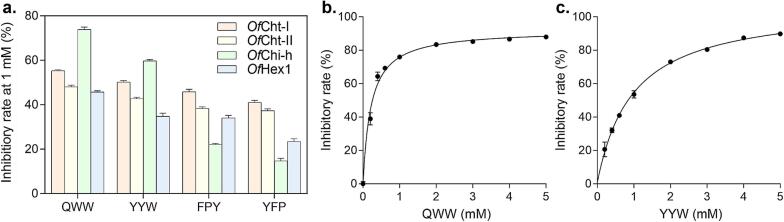
(a) Inhibitory rates of QWW, YYW, FPY, and YFP against *Of*ChtI, *Of*ChtII, *Of*Chi-h, and *Of*Hex1. (b) IC_50_ curve of QWW against *Of*Chi-h (IC_50_ = 0.21 mM). (c) IC_50_ curve of YYW against *Of*Chi-h (IC_50_ = 0.98 mM). Data are presented as mean ± standard deviation.

We evaluated the inhibitory activity of the selected tripeptides QWW, YYW, FPY, and YFP against four chitinolytic enzymes from *O. furnacalis*. Their broad-spectrum inhibitory efficacy was assessed at a fixed concentration of 1 mM (Fig. 3a). The results demonstrated that the inhibitory efficacy was strongly associated with the amino acid composition of the peptides and structural variations among the target enzymes.

Among the tested peptides, QWW demonstrated the most potent and broad-spectrum inhibitory activity, with inhibitory rates of 73.90 % against *Of*Chi-h and 55.29 % against *Of*ChtI. The presence of two tryptophan residues may contribute to its higher overall efficacy. YYW, which contains one tryptophan and two tyrosine residues, exhibited moderate inhibition on four enzymes, with an inhibitory rate of 59.79 % against *Of*Chi-h. In contrast, FPY and YFP displayed significantly weaker inhibitory effects, with all inhibitory rates below 50 %. YFP, in particular, showed the lowest activity, with only 14.76 % inhibition against *Of*Chi-h. The absence of tryptophan and the overall sequence composition may account for the reduced performance observed in these peptides.

To further quantify the inhibitory effects, we determined the IC_50_ values of QWW and YYW, both of which exceeded 55 % inhibition against *Of*Chi-h at 1 mM. The dose–response experiments revealed an IC_50_ of 0.21 mM for QWW (Fig. 3b), confirming its high potency, while YYW exhibited an IC_50_ of 0.98 mM (Fig. 3c), consistent with its moderate inhibitory profile. Although FPY and YFP did not achieve sufficient inhibition (>50 %) for reliable IC_50_ determination, their residual activity against *Of*ChtI and *Of*Hex1 suggests that phenylalanine and tyrosine may contribute to enzyme binding in certain conformational settings, albeit less effectively than tryptophan-containing analogues.

### 3.4 Binding mechanisms of tripeptides with *Of*Chi-h

**Fig. 4:**
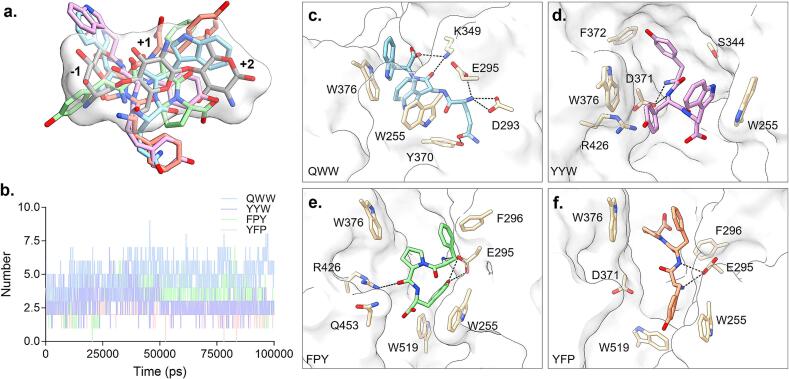
(a) Superimposed binding conformations of the tripeptides QWW, YYW, FPY, and YFP with the trisaccharide substrate in *Of*Chi-h, with the substrate surface rendered. The tripeptides QWW, YYW, FPY and TFP were shown as blue, pink, green, and orange, respectively. The trisaccharide substrate was shown as gray. (b) Number of hydrogen bonds formed during molecular dynamics simulations of the four tripeptides. Predicted binding conformations of QWW (c), YYW (d), FPY (e), and YFP (f) with *Of*Chi-h obtained from molecular dynamics simulations. Key interacting residues within the catalytic site are highlighted and displayed as sticks, with hydrogen bonds represented by dashed lines. (For interpretation of the references to colour in this figure legend, the reader is referred to the web version of this article.)

To investigate the structural determinants underlying the differential inhibitory activity of the four selected tripeptides (QWW, YYW, FPY, and YFP) against *Of*Chi-h, we first conducted molecular docking simulations using LeDock. The top-ranked binding poses of the tripeptides were superimposed with that of the native trisaccharide substrate, revealing that all four peptides occupied spatial regions adjacent to the substrate within the active site (Fig. 4a). Notably, QWW and YYW exhibited binding orientations more similar to the substrate and each other, while FPY and YFP displayed relatively distinct but mutually similar conformations. To further explore the stability and binding dynamics of these peptides, molecular dynamics (MD) simulations were performed based on their optimal docked conformations. During the final 30 ns of the simulation (Fig. S1), QWW and YYW maintained relatively stable binding poses, whereas FPY and YFP exhibited more pronounced structural drift, indicating weaker and less persistent association with the active site. Hydrogen bond occupancy analysis over the 100 ns MD trajectories revealed marked differences among the peptides, with QWW and FPY forming more extensive hydrogen bonding networks than YYW and YFP (Fig. 4b).

Subsequent structural analysis of the equilibrated binding conformations (Fig. 4c–f) revealed distinct interaction profiles across the four tripeptides. QWW formed multiple stabilizing interactions, including hydrogen bonds between its terminal carboxyl and backbone carbonyl groups with Lys^349^, and electrostatic interactions between its amino group and Glu^295^/Asp^293^. Additionally, its glutamine side chain engaged in hydrogen bonding with Tyr^370^. Most importantly, the two tryptophan residues formed parallel-displaced π–π interactions with Trp^255^ and Trp^376^, contributing significantly to complex stabilization.

In contrast, YYW established fewer polar contacts, primarily involving hydrogen bonding through its N-terminal amino and backbone atoms with Asp^371^, and π–π interaction between tyrosine and Phe^372^/Trp^376^ in T-shaped and offset-parallel modes. Similarly, FPY formed hydrogen bonds between its amino group and tyrosine hydroxyl with Glu^295^, and additional contacts with Arg^426^. Aromatic stacking was also observed between phenylalanine and Phe^296^ (T-shaped) and tyrosine with Trp^255^ (parallel-displaced). Conversely, YFP exhibited the weakest interaction profile, forming only minimal hydrogen bonds and a single T-stacking interaction between tyrosine and Trp^519^.

This comparative analysis highlights the cooperative role of dual aromatic residues and a flexible polar side chain in QWW, which promotes both π–π interactions and hydrogen bonding, thereby enhancing conformational adaptability. These dual interaction modes likely underpin QWW's broad-spectrum inhibitory activity.

To quantitatively support these findings, binding free energies were estimated using the molecular mechanics Poisson–Boltzmann surface area (MM/PBSA) method based on MD trajectories (Table 1, Fig. S2–S5). QWW exhibited the lowest total binding free energy (ΔG_total_ = −49.49 ± 2.11 kcal/mol), primarily driven by strong electrostatic interactions (ΔG_elec_ = −198.83 ± 1.64 kcal/mol) and favorable polar solvation contributions. YYW showed the strongest van der Waals interactions (ΔG_vdW_ = −45.05 ± 1.32 kcal/mol) due to its high aromatic content, yet its overall binding energy (ΔG_total_ = −37.53 ± 5.53 kcal/mol) was less favorable owing to weaker electrostatic stabilization. FPY demonstrated moderate binding affinity (ΔG_total_ = −32.94 ± 6.45 kcal/mol), with significant electrostatic contributions but lower van der Waals interactions. YFP yielded the least favorable binding profile (ΔG_total_ = −26.71 ± 4.46 kcal/mol), consistent with its minimal binding interactions observed in structural analyses.

**Table 1:** Binding free energy decomposition of tripeptide complexed with *Of*Chi-h.

Tripeptide	Binding free energy (kcal/mol)				
	ΔG_vdW_	ΔG_elec_	ΔG_polar_	ΔG_nonpolar_	ΔG_total_
QWW	−38.93 ± 1.12	−198.83 ± 1.64	193.96 ± 0.70	−5.69 ± 0.04	−49.49 ± 2.11
YYW	−45.05 ± 1.32	−32.53 ± 5.19	44.64 ± 1.39	−4.59 ± 0.05	−37.53 ± 5.53
FPY	−30.84 ± 0.34	−106.75 ± 4.53	109.34 ± 4.58	−4.69 ± 0.20	−32.94 ± 6.45
YFP	−40.02 ± 1.96	−52.44 ± 3.84	70.65 ± 1.04	−4.90 ± 0.43	−26.71 ± 4.46

Collectively, these results emphasize the critical role of balanced aromatic stacking, hydrogen bonding, and electrostatic interactions in determining tripeptide binding affinity and inhibitory potential toward *Of*Chi-h.

## 4 Discussion

This study introduces an innovative strategy for designing chitinase inhibitors by randomly assembling natural amino acids to construct a diverse library of 8000 tripeptides. These peptides were screened through virtual screening and experimental validation to identify those with significant chitinase inhibitory activity. This method offers clear advantages over traditional cyclic peptide inhibitors in terms of structural simplicity, synthetic feasibility, and scalability for large-scale production (Badreldin et al., 2024; Nandhini et al., 2023; Qiu et al., 2023).

Among the screened tripeptides, QWW exhibited the most potent inhibitory activity against four enzymes, with an IC_50_ value of 0.21 mM for *Of*Chi-h, demonstrating micromolar-range inhibition. Molecular dynamics simulations revealed that QWW forms stable hydrogen bonds and π-π stacking interactions with the chitinase binding pocket, primarily due to the aromatic properties of its tryptophan residues. These interactions enhance the stability and affinity of the enzyme-peptide complex, contributing to the peptide's high inhibitory activity. In contrast, other tripeptides such as YYW, FPY, and YFP exhibited weaker inhibitory activity, which can be attributed to differences in their amino acid compositions and structures. The study further highlighted that peptides enriched with aromatic amino acids and possessing higher hydrophobicity tend to exhibit stronger inhibitory activity, as these properties promote more stable interactions with the chitinase substrate-binding pocket. The combined effects of aromatic stacking and hydrogen bonding are crucial for the high efficacy of QWW.

Additionally, the study analyzed the general structural characteristics of chitinase inhibitors. Peptides enriched with aromatic amino acids and greater hydrophobicity tend to exhibit stronger inhibitory activity, as these properties enable more stable interactions with the substrate-binding pocket. Specifically, the interactions between aromatic amino acids facilitate tighter binding, while hydrogen bonds play a critical role in stabilizing the overall conformation of the peptide. For example, QWW optimizes its inhibitory efficacy through aromatic stacking and the synergistic effect of hydrogen bonds, ensuring a high degree of compatibility with the chitinase binding pocket. These structural features underscore the importance of carefully selecting amino acid composition and positioning to enhance binding affinity and stability, thereby improving inhibitory potency.

In conclusion, this research proposes a simple and scalable method for designing peptide inhibitors by assembling natural amino acids, expanding the chemical space for potential chitinase inhibitors. The simplicity and feasibility of this approach provide a clear advantage, offering a versatile platform for developing inhibitors that can be applied across various enzyme-targeting studies. This versatile strategy not only simplifies inhibitor design but also enables the efficient development of inhibitors for diverse applications.

## CRediT authorship contribution statement

**Zihan Pang:** Writing – original draft, Methodology, Investigation, Formal analysis, Data curation. **Yi Ding:** Writing – original draft. **Huijie Xie:** Formal analysis. **Xi Jiang:** Visualization, Investigation. **Tian Liu:** Writing – review & editing, Supervision, Project administration, Conceptualization.

## Declaration of competing interest

The authors declare that they have no known competing financial interests or personal relationships that could have appeared to influence the work reported in this paper.

## Appendix A Supplementary data

Supplementary figures. Image 1
