# Supplementary material for: Virtual screening of a random tripeptide library for easily prepared inhibitors of insect chitinolytic enzymes
Source: Cell Surf. 2025 May 13;13:100143. doi: 10.1016/j.tcsw.2025.100143 (PMC12143796; doi:10.1016/j.tcsw.2025.100143)
Supplement: Supplementary file 1 — Supplementary figures. [file mmc1.docx]

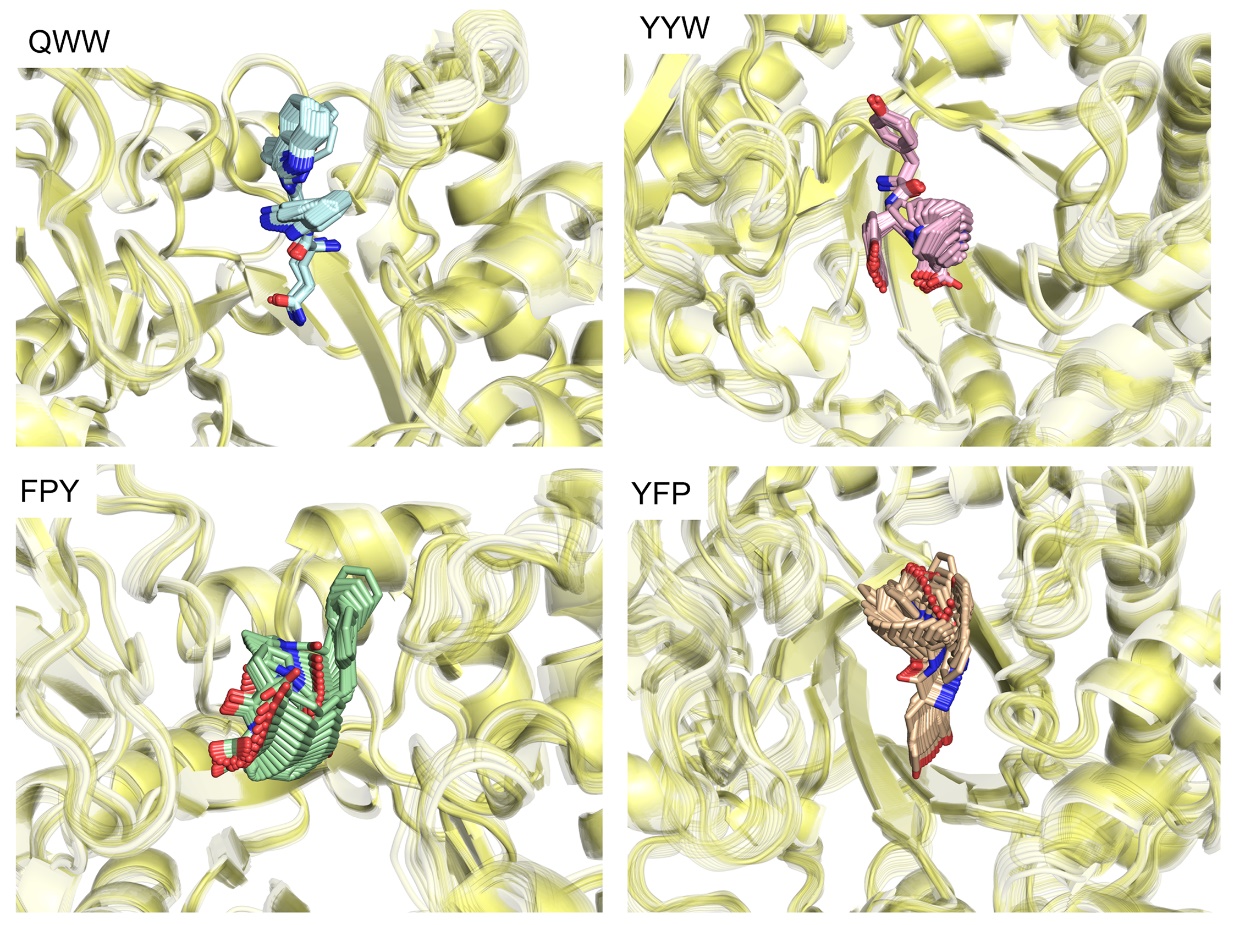


**Figure S1**. Superimposed conformations from the final 30 ns of molecular dynamics simulations. *Of*Chi-h is shown in yellow cartoon representation, while the four tripeptides are displayed as sticks: QWW (blue), YYW (purple), FPY (green), and YFP (brown). The figure illustrates the conformational stability and spatial positioning of each peptide within the binding site.


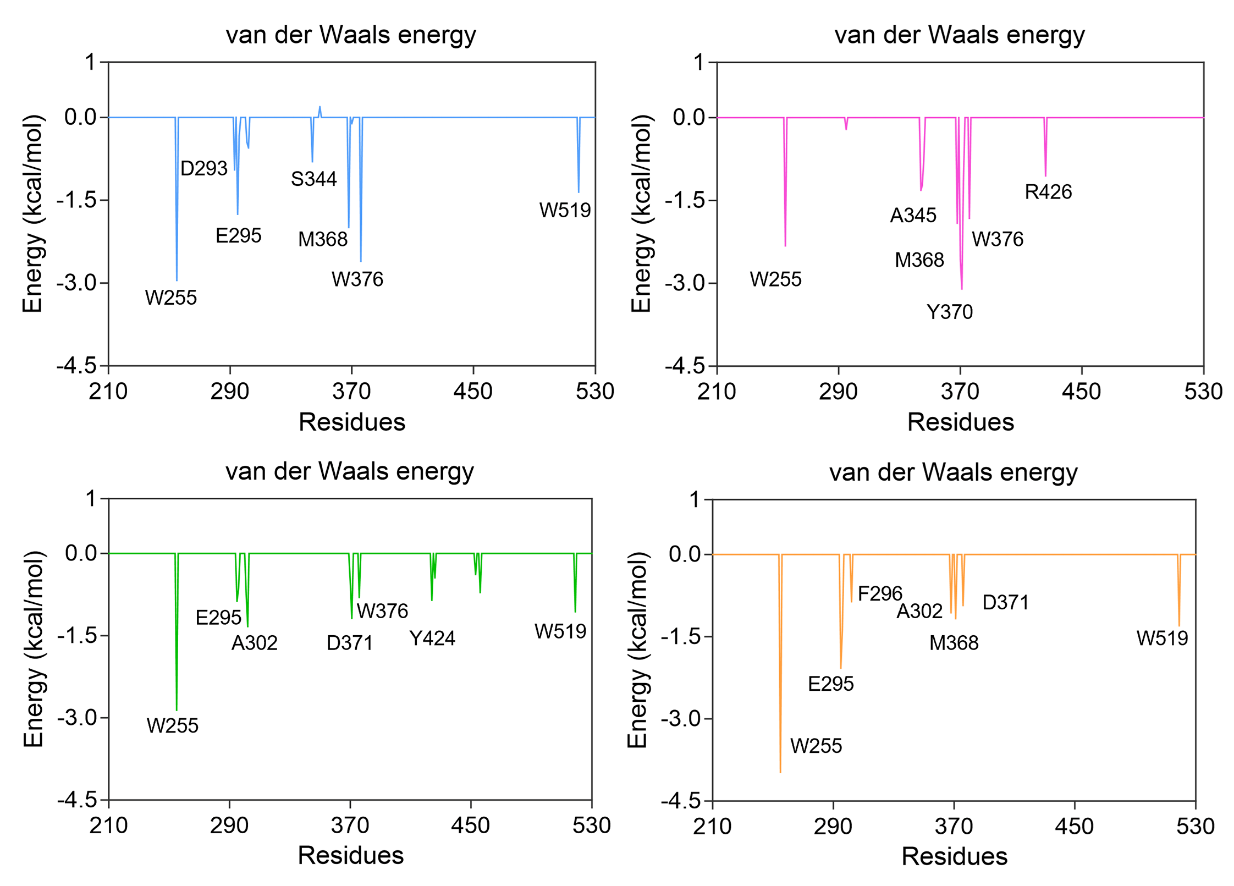


**Figure S2**. Van der Waals interaction energy contributions between tripeptides and *Of*Chi-h. The plots illustrate the van der Waals interaction energies for QWW (blue), YYW (purple), FPY (green), and YFP (orange) throughout the binding simulations with *Of*Chi-h. Negative values indicate favorable interactions, with more negative values suggesting stronger and more stable binding.


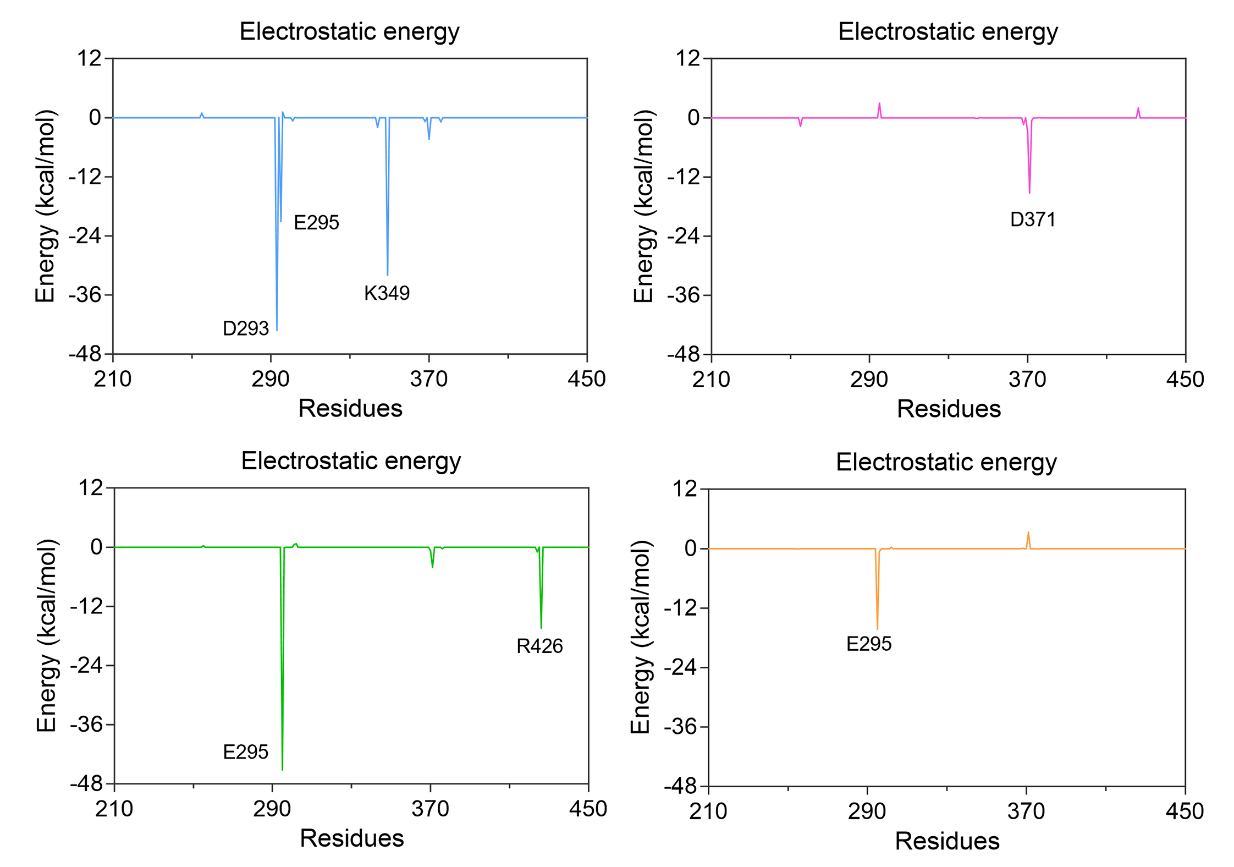


**Figure S3**. Electrostatic energy contributions between tripeptides and *Of*Chi-h. The plots display the electrostatic energy contributions of key residues during the interaction of QWW (blue), YYW (purple), FPY (green), and YFP (orange) with *Of*Chi-h. Negative values represent favorable electrostatic interactions.


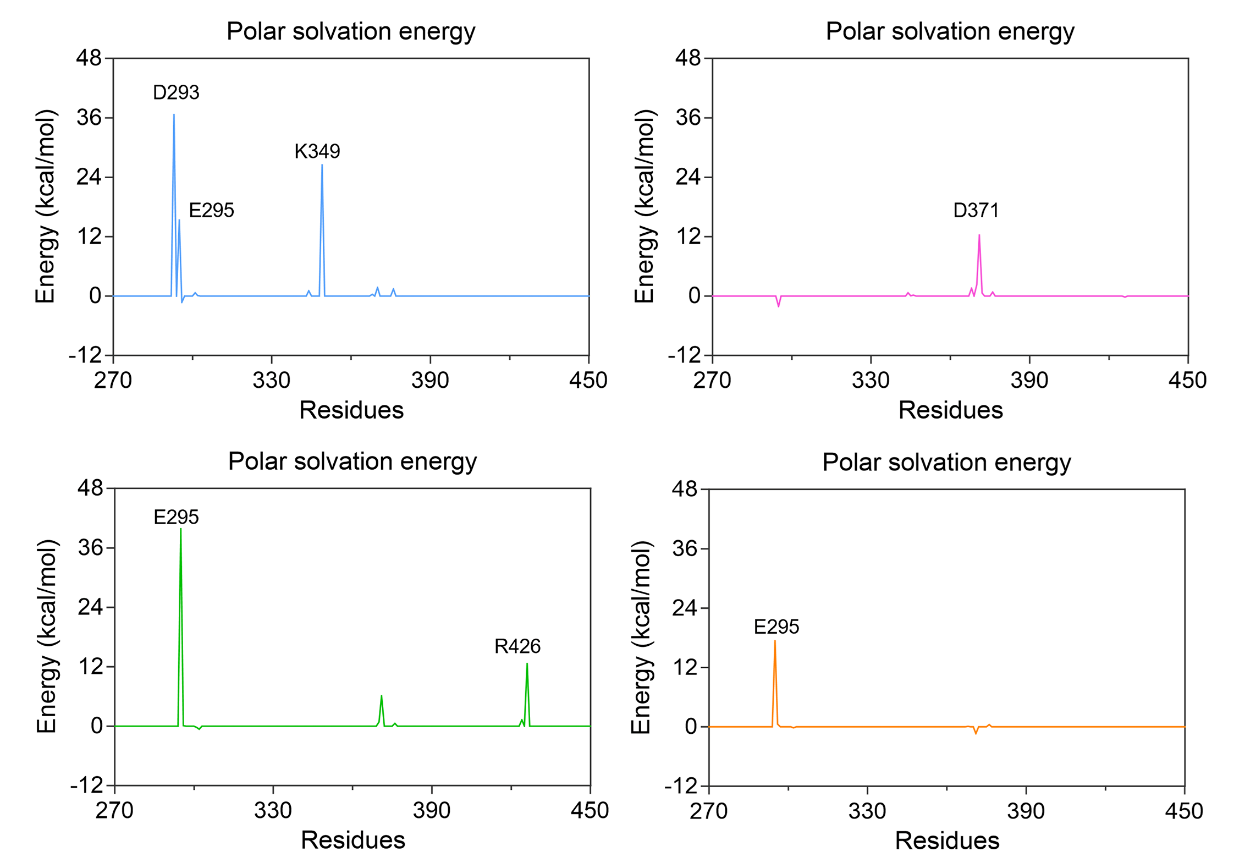


**Figure S4**. Polar solvation energy contributions between tripeptides and *Of*Chi-h. The plots display the polar solvation energy contributions for QWW (blue), YYW (purple), FPY (green), and YFP (orange) during binding to *Of*Chi-h. Positive values indicate unfavorable contributions to binding due to polar solvation effects.


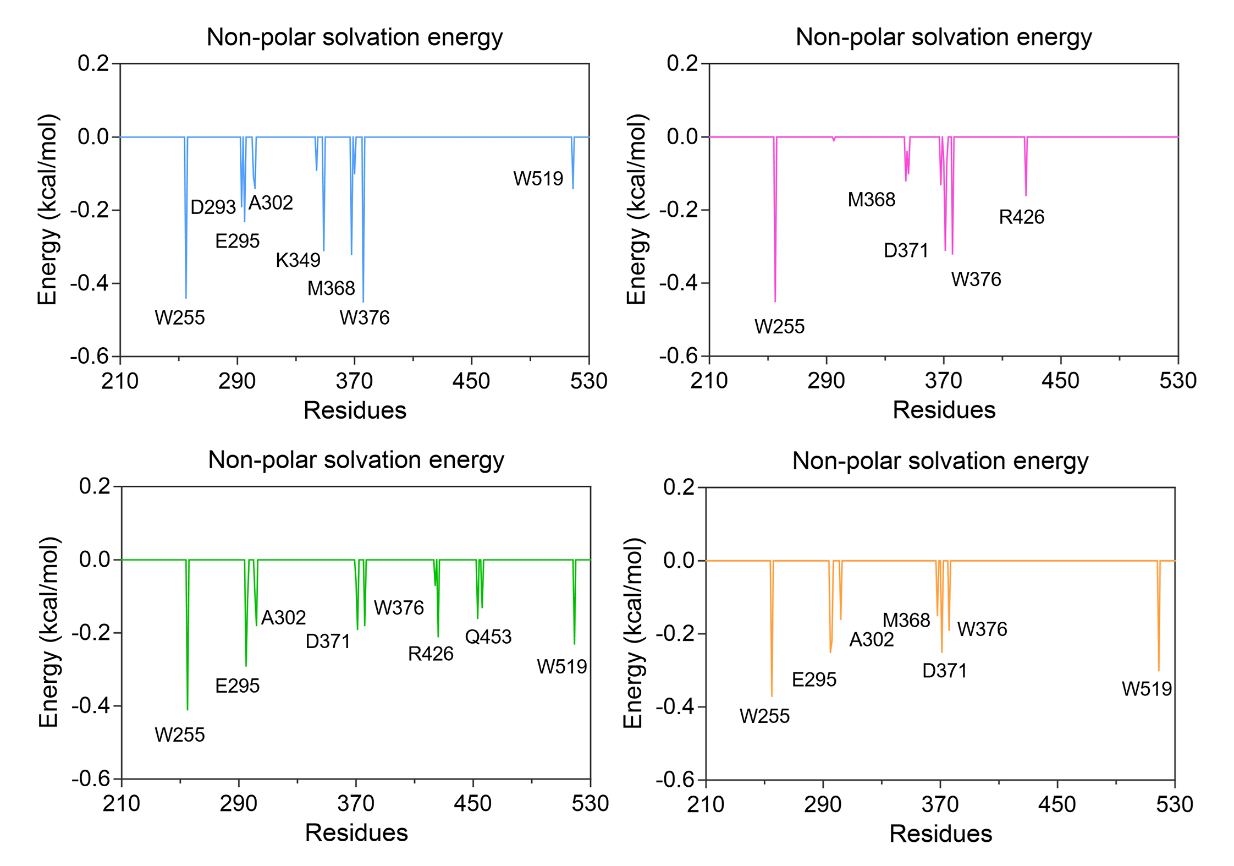


**Figure S5**. Non-polar solvation energy contributions between the four tripeptides and *Of*Chi-h. The plots display the polar solvation energy contributions for QWW (blue), YYW (purple), FPY (green), and YFP (orange) during binding to *Of*Chi-h. Negative values indicate favorable contributions to binding due to non-polar solvation effects.
